# Early Life Exposure to Fructose and Offspring Phenotype: Implications for Long Term Metabolic Homeostasis

**DOI:** 10.1155/2014/203474

**Published:** 2014-04-23

**Authors:** Deborah M. Sloboda, Minglan Li, Rachna Patel, Zoe E. Clayton, Cassandra Yap, Mark H. Vickers

**Affiliations:** ^1^The Department of Biochemistry and Biomedical Sciences, McMaster University, 1280 Main Street West, HSC 4H30A, Hamilton, ON, Canada L8S 4K1; ^2^The Liggins Institute and Gravida: National Centre for Growth and Development, University of Auckland, Auckland 1142, New Zealand

## Abstract

The consumption of artificially sweetened processed foods, particularly high in fructose or high fructose corn syrup, has increased significantly in the past few decades. As such, interest into the long term outcomes of consuming high levels of fructose has increased significantly, particularly when the exposure is early in life. Epidemiological and experimental evidence has linked fructose consumption to the metabolic syndrome and associated comorbidities—implicating fructose as a potential factor in the obesity epidemic. Yet, despite the widespread consumption of fructose-containing foods and beverages and the rising incidence of maternal obesity, little attention has been paid to the possible adverse effects of maternal fructose consumption on the developing fetus and long term effects on offspring. In this paper we review studies investigating the effects of fructose intake on metabolic outcomes in both mother and offspring using human and experimental studies.

## 1. Dietary Trends in Today's Society

The prevalence of obesity and diabetes has increased radically on a global scale over the past two decades to a point where these conditions are now considered to be “epidemic.” There are an estimated 312 million obese adults worldwide and at least 155 million children who are either overweight or obese [1]. This problem is not limited to developed countries; rates of obesity have tripled in developing countries as they adopt a western lifestyle characterised by less physical activity and over indulgence in high-calorie processed foods [1, 2]. The increase in obesity has been accompanied by an increase in the incidence of type 2 diabetes, particularly in areas that have undergone fairly rapid urbanisation [3, 4]. Advances in food processing technologies have significantly contributed to an influx of inexpensive, energy dense diets [5] that are readily available worldwide.

Countries tend to undergo nutritional transitions from traditional grain-based diets to high-fat high-sugar diets as affluence increases and populations become more urban [6]; countries with the highest incomes have the highest fat and sugar intakes [5]. Throughout the world, the consumption of fat and sugar is on the rise; their availability per capita has increased by more than 20% since 1977 and the consumption of nonsucrose caloric sweeteners has also seen an upsurge since 1962 [5, 7, 8]. Increasing attention is being paid to the large increase in sweetened beverage consumption worldwide which has been primarily driven by increased sweetened beverage intake which makes up a substantial proportion of the total increased caloric load in many developed countries [9]. Shifts in the use of fructose and high fructose corn-syrup from sucrose in sweetened beverages as well as in processed foods have been suggested to play a role in the obesity trend [10–13], although in some populations sugar intake may be on the decline [14]. Although evidence exists suggesting that excessive fructose intake could be one determinant in the aetiology of the metabolic syndrome long term impaired hepatic pathophysiologies (Nonalcoholic Fatty Liver Disease, NALFD) and type 2 diabetes [11, 15, 16], other studies suggest that this relationship is not quite as straightforward as some believe [17, 18]. Currently, although the experimental data are clear, there is a significant paucity of well designed, adequately powered human studies to fully investigate the long term effects of fructose intake on metabolic function. Despite this, a report of the 8th Annual World Congress on Insulin Resistance, Diabetes, and Cardiovascular Disease highlighted the role of excessive fructose intake in the worldwide increasing prevalence of type 2 diabetes, obesity, and their comorbidities [19]. However, a recent meta-analysis suggests that the association between fructose intake and dyslipidemia occurs only when fructose intake is excessive and in positive energy balance [20]. Thus, the controversy regarding fructose intake and long term effects in humans still points toward the need for large appropriately powered randomized controlled trials in humans.

## 2. Fructose: What Is It and How Much Do We Consume?

Fructose is a naturally occurring sugar and the predominant monosaccharide found in honey, fruit, and some vegetables [21]. It is a component of the disaccharide sucrose, formed by condensation of fructose and glucose monomers [22]. Fructose is a 6-carbon polyhydroxyketone and is an isomer of glucose; it has the same molecular formula but differs structurally. Fructose can be produced from glucose by isomerisation, a reaction which is utilised in the production of high fructose corn syrup (HFCS) [23].

In recent decades, human consumption of free fructose has increased with the introduction of both fructose and HFCS as manufactured sugars in many soft drinks and processed foods. It is now estimated that the average American consumes approximately 50 g of fructose on a daily basis [24] and since 1978 mean daily intakes of added and total fructose increased in all gender and age groups, whereas naturally occurring fructose intake decreased or remained constant [25]. Fructose is more than twice as sweet as glucose and more than 1.5 times sweeter than sucrose [26] when sucrose is used as a baseline measure of sweetness [27]. It is also more soluble in water, which makes it an ideal sweetener for beverages and tinned fruits [27, 28]. HFCSs were first introduced in 1967 and levels of consumption increased by more than 1000% between 1970 and 1990 [26]. The increase in HFCS consumption has been particularly marked in the United States, where HFCS now accounts for more than 40% of caloric sweeteners added to food and beverages [26].

A National Health and Nutrition Examination Survey (NHANES III), conducted in the United States between 1988 and 1994, included dietary fructose intake as a reported variable and its recent analysis found that on average fructose accounted for over 10% of daily caloric intake in adults and over 12% of daily caloric intake in adolescents, with a quarter of adolescents obtaining at least 15% of their caloric intake from fructose [24]. The major source of fructose was found to be sugar-sweetened beverages, which accounted for over 30% of fructose consumed; a further 21.5% came from grains, a category which includes processed foods, such as cakes, breads, and pies. Fructose from fruit in the form of fruit juices accounted for just 19.1% of fructose intake. Given the continued increase in the use of fructose and HFCSs and the inconsistencies in reporting fructose intake, it has been suggested that the percentage of energy consumed as fructose is greater than 20% in a substantial proportion of the US population [24]. The most recent analysis from the National Health and Nutrition Examination Survey 2005–2008 reported that one half of the US population consumes sugar drinks on any given day and that 25% consumes at least 200 kcal, which is equivalent to more than one 12 oz can of soft drink and as much as 5% of the population consumes at least 560 kcal, more than four 12 oz cans of soft drink [29]. Not surprisingly, in 2010, the US Dietary Guidelines for Americans policy document recommended a reduction in the intake of added sugars in the diet to reduce overall calorie intake [30].

HFCS may be present as different formulations consisting of a mixture of glucose and fructose. Commercial HFCS typically contains either 42% (HFCS-42) or 55% (HFCS-55) fructose mixed with glucose. This is biochemically different from sucrose in that fructose and glucose in HFCS are present in monosaccharide form rather than linked by a glycosidic bond to form a disaccharide [23, 26, 31] and therefore has different metabolic properties. Nonetheless, the consumption of glucose in addition to fructose is important to consider, as it has been shown to modulate both the absorptive and metabolic properties of fructose [21, 32, 33].

## 3. Biochemistry of Fructose—What Makes It Different from Sugar

In the past, the characteristics of fructose absorption were not of particular interest because dietary fructose levels were relatively low. However, because of its commercial value, the significant introduction of fructose and HFCSs in processed foods and beverages, and the resulting increase in human consumption of free fructose, understanding the mechanisms of fructose absorption became increasingly important. Initially, rodent studies established transporter mechanisms showing that an active carrier-mediated mechanism of fructose uptake was present in rat small intestine [22, 34]. Studies on the absorption kinetics of free fructose in rodents found that fructose transport across the intestinal epithelial membrane may be a partially active process, as the rate of transport falls between that of actively transported sugars, such as glucose, and the rate of passively absorbed sugars [28]. However, no evidence of active transport of fructose has been found in human studies and it is generally accepted that intestinal absorption of fructose in humans occurs via facilitated diffusion alone, through members of the glucose transporter family, GLUT5 and GLUT2 [22], although GLUT 5 is the sole transporter specific for fructose with no ability to transport glucose or galactose [35].

Studies investigating acute responses to both sucrose and HFCS have found similar effects on glucose, insulin, leptin, and ghrelin profiles between the two sweeteners, which were intermediate between those induced by fructose alone and those induced by glucose alone [36]. Both sucrose and HFCS increased plasma triglycerides to levels comparable to those induced by fructose alone and it has been suggested that the lipogenic properties of sucrose and HFCS can be attributed to their fructose component [36]. A dose-dependent effect of glucose on fructose absorption capabilities has been demonstrated [37]. Consumption of a mixture of fructose and glucose has been shown to significantly increase the absorptive capacity for fructose and eliminate signs and symptoms of malabsorption [32]. This facilitative phenomenon is thought to be the result of apical insertion of GLUT2 transporters in response to sodium-glucose linked transporter 1 (SGLT1) transport of glucose, enabling more efficient uptake of all hexoses [38, 39]. This ability of glucose to enhance fructose absorption has potentially significant metabolic implications, given that fructose is often consumed in conjunction with glucose as either sucrose or HFCS. In addition, utilisation of fructose by the liver is altered by the presence of glucose where fructose is preferentially directed through glycogenic and lipogenic pathways [28, 33].

Following an acute dose of fructose, the increased availability of intermediates in the glycolytic pathway leads to increased production of pyruvate and lactate, the end products of glycolysis [33]. Importantly, fructose consumption does not lead to pancreatic insulin secretion due to an absence of GLUT5 transporters in the pancreas and the inability of fructose to stimulate gastric inhibitory peptide, which stimulates insulin secretion [26]. Acute fructose consumption also fails to stimulate leptin release from adipose tissue [40]; as a result plasma levels of glucose, insulin, and leptin are lower in the 24-hour period following consumption of fructose or fructose-sweetened beverages, compared with glucose or glucose sweetened beverages [40, 41]. In the postprandial period, fructose also fails to suppress the release of ghrelin, a potent appetite stimulating peptide [40]. However, fructose has been shown to stimulate the release of gastric leptin, which in turn leads to increased insertion of GLUT 2/5 transporters into the apical membrane of the small intestine and increased fructose transport across the intestinal wall [42]. It appears that at least biochemically the actions of fructose have potentially negative consequences on overall metabolic function if fructose is consumed excessively for long periods of time.

## 4. Fructose Effects: Data from Experimental Studies in Animals

Much of what is known about the long term effects of fructose has come from experimental studies in rodents. The fructose-fed rat is an animal model of acquired systolic hypertension and the metabolic syndrome [43]. Early studies investigating high doses of fructose (50–60%) reported that rats consuming a high-fructose diet developed insulin resistance, hypertension, and hypertriglyceridaemia [44]. Consistent with this, fructose-fed rats demonstrated diminished suppression of hepatic glucose outflow in response to insulin, characteristic of hepatic insulin resistance [45]. Chronic fructose consumption (60.4% kcal derived from fructose) in the rodent did not alter food intake or weight gain but induced leptin resistance, which exacerbated weight gain in response to a high fat diet [46]. Other studies have shown that the hepatic effects of fructose can be achieved in rodents using diets with as little as 10% wt/vol of fructose in drinking water [47–49]. However, one must note that often studies [46, 48, 49] do not account for an overall reduction in food intake; thus, the total calories derived from fructose in these studies are likely higher than the reported 10%. Hence, while one can commonly observe similar overall energy intakes between control and fructose groups, when one looks at where the animals are getting energy from a different story may unfold. Thus, interpretation of experimental studies requires both the consideration of delivery mode and total relative percentage of calories derived from fructose, which is often not delineated in published experimental studies.

Numerous pathways have been proposed to mediate the effects of fructose, including uric acid formation, inflammation, leptin resistance, and advanced glycation end products [43, 50–52]. Although the precise mechanisms remain uncertain, these findings experimentally and biochemically implicate consumption of fructose in the development of metabolic compromise.

## 5. Fructose Effects: Data from Human Studies

In humans, it can be difficult to isolate the effects of chronic fructose consumption, particularly over longer periods of time. Responses to a single dose of fructose alone are likely to be different from those elicited by a meal containing fructose and different again from those elicited by chronic fructose intake [28]. Furthermore, human studies suffer from issues with diet composition, subject compliance, and individual variation in responses [28]. To date there is not a clear consensus on the long term effects of fructose intake in humans; studies are limited by sample size and power analysis and are often retrospective.

Of the few studies that have investigated the effects of fructose consumption in human populations, many show changes related to lipid regulation. Fructose is more lipogenic than glucose and its consumption typically results in immediate elevations in plasma triglycerides, despite the fact that it provides substrates for gluconeogenesis and glycogenesis. Fructose consumption favours triglyceride synthesis, particularly when glucose is also present. Studies of acute fructose intake have consistently observed elevated plasma triglycerides, and sometimes VLDL, in the 24 h period following a dose of fructose or a fructose-sweetened beverage [36, 53, 54]. Fructose bypasses the rate-limiting step of glucose metabolism and provides unregulated amounts of lipogenic substrates for conversion to fatty acids and triglycerides as well as upregulating transcription factors and enzymes involved in lipogenesis, including sterol regulatory element binding protein 1c (SREBP1c) and acetyl-coenzyme A carboxylase (ACC) [36]. It has been suggested that attenuated stimulation of insulin secretion after fructose may result in reduced activation of adipose tissue lipoprotein lipase, leading to impaired triglyceride clearance [53].

Fructose consumption also leads to hyperuricemia [55]. The conversion of fructose to fructose-1-phosphate depletes hepatic ATP stores, activating enzymes involved in the degradation of AMP and formation of uric acid [33]. Hyperuricemic responses have been observed in subjects who consumed approximately 10% of their energy intake as fructose, which suggests that an average individual's intake of fructose is on the borderline for inducing hyperuricemia [33]. Chronic elevation of plasma uric acid levels has been linked to endothelial dysfunction and impaired insulin action via a reduction in endothelial nitric oxide (NO) [50]. Coupled with the observation that increased uric acid concentrations correlate with the increasing prevalence of the metabolic syndrome, these data suggest that fructose-induced hyperuricemia has a pathogenic role in hypertension, insulin resistance, and obesity [16, 50]. Reductions in insulin secretion and attenuated 24 h leptin profiles observed in short-term studies are not transient but are maintained after long term fructose consumption [36]. Long term consumption of a high-fructose diet also leads to hepatic enzyme adaptation and the upregulation of fructose-1-6-bisphosphatase, glycogen synthase, glucose-6-phosphatase, and lipogenic enzymes. As a result, more fructose is converted to glucose and glycogen, there is increased long chain fatty acid synthesis and hepatic triglyceride, and very low density lipoprotein (VLDL) output is increased, which leads to hypertriglyceridaemia [33].

## 6. Perinatal Fructose Intake

Western populations obtain >10% of their daily caloric intake from fructose [56]; thus, the long term consequences of maternal fructose consumption are relevant. The major contributors to the total energy intake in women during pregnancy have been reported to consist of low-nutrient-energy dense foods, comprising high levels of refined carbohydrates and saturated fat [57] and are particularly prevalent in low income populations [58]. High maternal intake of nutrient-poor energy-dense foods may suggest that in many pregnancies the nutritional needs of a healthy growing fetus may be compromised. Indeed recent studies show that few women succeed in complying with nutrition and lifestyle recommendations for planning a pregnancy [59] and only 46% of women in the Southampton Women's survey achieved the recommended intake of fruits and vegetables in early pregnancy [60].

It is well established that fructose is an important energy source during fetal life, particularly in ruminants [61, 62]; in the ovine placenta glucose is used in the production of endogenous fructose [62] and fructose is found in the fetal circulation in significant amounts, although for the most part glucose is the primary substrate for oxidative metabolism [61, 63]. It has been suggested that the presence of fructose in human umbilical cord blood is indicative of fructose production by the fetal-placental unit [64]. Indeed, appreciable amounts of fructose have been found in first trimester amniotic fluid [65], similar to that observed in the sheep. Early work suggests that fructose is transported across the human placenta [66] and that the human placenta is capable of producing endogenous fructose [67]. The transport of fructose in other tissues is carrier mediated but there are little conclusive data identifying the presence of a GLUT5 transporter in human placental tissue. Although there is some evidence that placental transport of fructose may be diffusion mediated [68], recent localisation of GLUT9 in human placental tissue [69] may be indicative of another route of carrier mediated fructose transport.

## 7. Perinatal Fructose Intake: Human Trends and Outcomes

Due to its inability to stimulate an insulin response, fructose was historically suggested as an alternative fuel source in diabetics [70]. Early studies demonstrated that maternal fructose infusion during labor elevated blood sugars compared to control (no infusion) or an infusion of glucose and increased levels of pyruvate and lactate in newborns [71] and increased the degree of acidosis in the mother [72]. Work by Trindade et al. examined the presence of fructose in umbilical cord blood of full term newborns in low risk pregnancy. Fructose concentration in umbilical cord blood was higher than maternal blood, suggestive of endogenous fructose production by the fetal-placental unit. Furthermore, fructose concentrations were higher in newborns at 48 h after birth than in the fetal umbilical cord blood, suggesting that fructose production is a continuous process from fetus to newborn. It was proposed that fructose production by the sorbitol pathway, present in the fetus and newborn, is an alternative pathway in glucose metabolism that may be used to maintain redox balance in the fetus; thus, the route for metabolizing fructose is already present in the early steps of human development [64].

To date, there remains a paucity of data on the effects of dietary fructose intake during pregnancy and none that have examined long term effects on the offspring. What little evidence exists has been done using small animal studies.

## 8. Perinatal Fructose Intake: Evidence from Animal Models

In early animal studies, female rats fed a 50% fructose diet during pregnancy and lactation demonstrated significantly elevated plasma glucose concentrations and elevated liver gluconeogenic enzymes during pregnancy [73]. In early pregnancy, fructose-fed dams had elevated plasma triglycerides compared to control and sucrose fed dams, and pups born to fructose-fed dams exhibited hyperglycemia at birth but the fructose intake in this study clearly exceeded that of normal human intake [73].

Work by Ching et al. showed that a maternal 60% fructose diet led to increases in serum triglycerides, FFAs, and insulin in offspring. This was concomitant with elevated hepatic triglyceride concentrations, increased expression of acetyl-coenzyme A carboxylase beta (ACC2) and carnitine palmitoyltransferase (CPT1a), and decreased expression of antioxidant enzymes and fatty acid binding protein, PPAR-gamma coactivator 1-*α*, and peroxisome proliferator-activated receptor-*α* [74].

Zou et al. also used a supraphysiological fructose diet (63%) to show that maternal fructose intake results in fatty liver and glucose intolerance during pregnancy and lactation; although birth weights were not affected, weaning weights were significantly reduced in offspring of fructose fed dams [75]. However, studies in rodents using very high fructose diets are commonly associated with significant reductions in overall food intake and thus may be confounded due to macronutrient imbalances which are known independently to confer an increased risk of disease in offspring. There is also evidence that dietary fructose can perturb calcium (Ca^2+^) handling, particularly during physiologically challenging conditions such as lactation, due to fructose induced reductions in synthesis of 1,25-OH_2_D_3_ [76].

Most recently, it was demonstrated that the combination of chocolate and fructose consumption in pregnant dams resulted in increased total body fat and impaired liver function in offspring [77]. However, as with the Zou et al. study above, this work is potentially confounded by the fact that the mothers significantly reduced intake of the regular chow diet and as a result were protein deprived during pregnancy, a circumstance known to independently result in metabolic reprogramming of offspring [78].

Studies using lower fructose exposures have also reported significant changes in genes related to fatty acid biosynthesis and carbohydrate metabolism. Mukai et al. reported that a 10% fructose intake during rat pregnancy leads to the upregulation of maternal and fetal hepatic sterol regulatory element-binding protein- (SREBP-) 1c [79]. More recent work by Rodríguez et al. has shown that 10% fructose leads to maternal hypertriglyceridemia and altered maternal and fetal leptin signalling [80]. A further study also using a 10% fructose solution found that dams given fructose were hyperglycaemic, hyperinsulinemic, and hypertriglyceridemic and had heavier livers and higher body fat content and hepatic glycogen content than their control counterparts [81]. These offspring had lower birth weights and were hypoglycemic and hyperinsulinemic at weaning [81]. Alzamendi and colleagues found that male offspring of rats who received 10% fructose during lactation had increased body weights and increased food intake and were hyperinsulinemic at postnatal day 60 compared to control offspring [82]. These rats also demonstrated neuroendocrine dysfunction, with decreased hypothalamic responsiveness to leptin and decreased hypothalamic expression of anorexigenic peptides [82].

**Figure 1:**
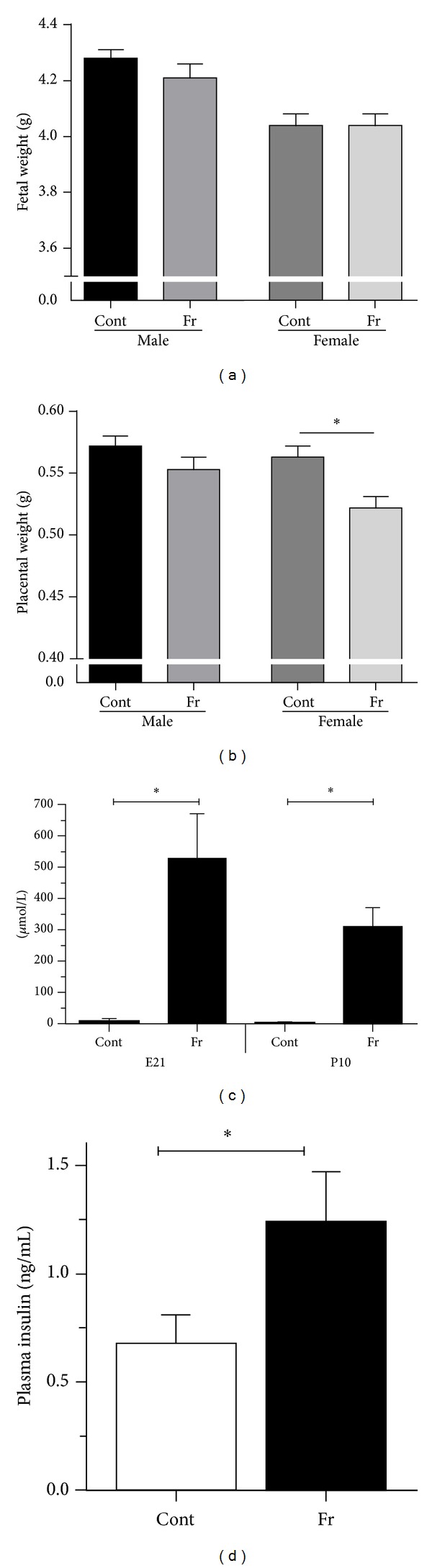
Fetal weight (a) and placental weight (b) at fetal day 21 (E21) and maternal plasma fructose levels at E21 and P10 (c) and maternal plasma insulin levels in Cont and Fr dams at E21 (d). Maternal fructose intake had no effect on fetal weight but significantly decreased placental weight in female fetuses, *P* < 0.05. Sample size: Cont male  (*n* = 10), Fr male  (*n* = 9), Cont female  (*n* = 10), and Fr female  (*n* = 9). Maternal fructose intake significantly increased maternal fructose at E21 and P10 and insulin levels at P10. Data are means ± SEM; *n* = 9-10 per group (E21) and *n* = 5–8 per group (P10). *P < 0.05. Cont = control; Fr = fructose. Data from Vickers et al. (2011).

We have showed that maternal intake of fructose (20% of total caloric intake) during pregnancy results in significant elevations in circulating maternal fructose levels accompanied by maternal hyperinsulinemia. One of the most important and novel observations of this study was the marked sex-specific effects of maternal fructose intake on placental growth and fetal and neonatal metabolic profiles. We demonstrated in term rat fetuses that maternal fructose intake significantly elevated circulating plasma leptin levels in females [83] and that these changes were paralleled by a reduction in female placental weights without any effect on fetal growth (Figure 1).

## 9. Countering the Adverse Metabolic Effects of Fructose

Several studies have examined possible intervention strategies to negate the adverse metabolic effects associated with excess fructose consumption. One of the most studied interventions is that of the amino acid taurine with a number of studies reporting beneficial effects of taurine supplementation in the setting of fructose-induced metabolic disorders [84–87]. Taurine has been shown to prevent hypertension and increase exercise capacity in rats with fructose-induced hypertension, possibly via antioxidation and maintenance of nitric oxide concentrations [88, 89]. Using a model of 25% fructose intake, it has been shown that taurine and L-arginine have synergistic effects on attenuating insulin resistance hypertension [90]. We have shown that taurine (2% in drinking water) can reverse the adverse metabolic effects in mother and offspring associated with a high fat : high fructose intake and also have data that maternal hyperinsulinemia associated with fructose intake is normalised with taurine supplementation [91]. However, as reported by Larsen et al., the combination of taurine and fructose can also increase fasting glucose levels in certain experimental models, therefore suggesting that the beneficial effect of taurine supplementation towards amelioration of glucose intolerance and insulin resistance may be dependent upon the dose and duration of fructose intake [92].

Resveratrol supplementation in the rat has been shown to restore many features of HFCS-induced disturbances including amelioration of vascular insulin resistance and endothelial dysfunction, possible by regulating eNOS and iNOS production [93, 94]. Further, a clinical trial with grape-derived polyphenols has also shown beneficial effects in prevention of fructose induced oxidative stress and insulin resistance in healthy volunteers with a high metabolic risk [95]. Dietary sardine protein can lower insulin resistance, leptin, and TNF-*α* and beneficially affect adipose tissue oxidative stress in rats with fructose-induced metabolic syndrome [96]. Supplementation of a high fructose diet with Vitamin E and *α*-lipoic acid ameliorates adverse cardiovascular and metabolic outcomes, possible via a reduction in lipid peroxidation [97, 98]. However, there remains a paucity of data regarding effective intervention strategies to negate the adverse metabolic effects of high fructose intake during critical developmental windows and effects on both mother and offspring.

## 10. Why Is This Important? 

Currently 36 million people die each year from noncommunicable diseases (NCDs) [99]. Obesity is a predisposing factor in chronic disease and is associated with a loss in life expectancy; in Canada having diabetes at age 55 has a loss of ~5 years in life expectancy [100]. As the rate of NCDs increases, the burden on health care will be unsustainable. Chronic disease risk is inextricably linked to our early life environment [101] where paternal, maternal, fetal, and childhood factors predict NCD risk later in life [101].

Almost two thirds of women of reproductive age in the United States are overweight translating to a marked increase in women entering pregnancy either overweight or obese. In 2006, >30% of Canadian women entered pregnancy either overweight or obese [102] and maternal obesity is the most significant predictor of childhood obesity [103] and metabolic complications in offspring [104]. Childhood obesity has become a global epidemic. In the US, the 2007-2008 National Health and Nutrition Examination Survey (NHANES) data showed that 17 percent of children and adolescents (ages 2–19 years) were obese, and over 30 percent were overweight or obese [105]. Nutrition and other prenatal environmental signals interact with the fetal genome impacting adult pathophysiology and chronic disease [106–108]. Prenatal signals, including nutrition, modulate disease risk by inducing integrated gene-environment interactions in fetal homeostasis leading to persistent changes in key metabolic signalling pathways. This, combined with an increased incidence of maternal obesity, has resulted in a feed-forward cycle of obesity/chronic disease where daughters of obese mothers are metabolically compromised becoming obese mothers themselves and give rise to another generation of children at risk of metabolic disease. Insulin resistance in children of obese mothers is present during fetal life and high maternal weight is associated with abnormal fetoplacental function [109], supporting the notion that lifelong metabolic health is determined very early in life.

Current evidence in experimental animal models strongly suggests that high fructose intake during pregnancy and lactation leads to metabolic dysfunction in both the mother and the newborn. Increased liver weight and glycogen content and the presence of hyperinsulinemia and elevated plasma glucose and triglycerides may suggest that fructose can promote lipogenesis and induce a state of hepatic insulin resistance in the mothers. Although offspring appear to have early onset changes in metabolic regulation it is unclear whether phenotypic effects in the offspring are the direct effect of fructose transfer through the placenta to the fetus, the effect of fructose transmitted to the neonate during lactation through the mother's milk, or the result of adaptive responses made by the offspring in response to altered maternal metabolism. It is clear that further research is required to elucidate the mechanisms behind the effects of maternal fructose intake on the mother and the offspring and to determine consequences for the offspring in adulthood.

While experimental investigations into the effects of excessive fructose intake (and that of other manufactured sugars) on metabolic function and obesity have shown clear long term compromise, whether the same effects hold true in human studies is still contentious. No doubt this is fuelled by the complexity of the human diet, owing to increased intake of overall calories derived from not only excessive sugar intake but high fat intake as well, thus making assessment of the direct effects of fructose* per se* difficult to delineate. The next few years will likely result in more cohort studies that are well-designed and controlled and which can effectively measure the impact of increased intake of manufactured sugars on metabolic outcomes in both mother and offspring.

Only recently has excessive fructose intake become the focus of perinatal research; as maternal obesity and excessive weight gain during pregnancy continue to rise it is now imperative that we begin to understand not only maternal global nutritional impacts on fetal, placental, and offspring development but also specific components of today's diet. Together with controlled studies of maternal nutrition during pregnancy in women, we will begin to elucidate the mechanisms of action where a particular focus should be on sex-specific effects and on placental function.
